# Brain networks supporting perceptual grouping and contour selection

**DOI:** 10.3389/fpsyg.2014.00264

**Published:** 2014-04-04

**Authors:** Gregor Volberg, Mark W. Greenlee

**Affiliations:** Institute of Psychology, University of RegensburgRegensburg, Germany

**Keywords:** EEG, contour integration, lateral occipital cortex (LO), ERP, beta oscillation

## Abstract

The human visual system groups local elements into global objects seemingly without effort. Using a contour integration task and EEG source level analyses, we tested the hypothesis that perceptual grouping requires a top-down selection, rather than a passive pooling, of neural information that codes local elements in the visual image. The participants were presented visual displays with or without a hidden contour. Two tasks were performed: a central luminance-change detection task and a peripheral contour detection task. Only in the contour-detection task could we find differential brain activity between contour and non-contour conditions, within a distributed brain network including parietal, lateral occipital and primary visual areas. Contour processing was associated with an inflow of information from lateral occipital into primary visual regions, as revealed from the slope of phase differences between source level oscillations within these areas. The findings suggest that contour integration results from a selection of neural information from lower visual areas, and that this selection is driven by the lateral occipital cortex.

## Introduction

It is a fundamental principle in primate vision that visual input is fragmented and processed within specialized brain modules (Livingstone and Hubel, 1988). This processing is accomplished by neurons with small receptive field sizes at the initial stages of the visual stream. Thus, parts of the same object are coded within different cells if the object extends beyond the receptive field size of an individual neuron (Roelfsema, 2006). In this case, an integration across space is necessary for assigning the object parts to a common object (Robertson, 2003). This integration of local parts into a global whole in visual perception is the general topic of this study.

**Figure 1:**
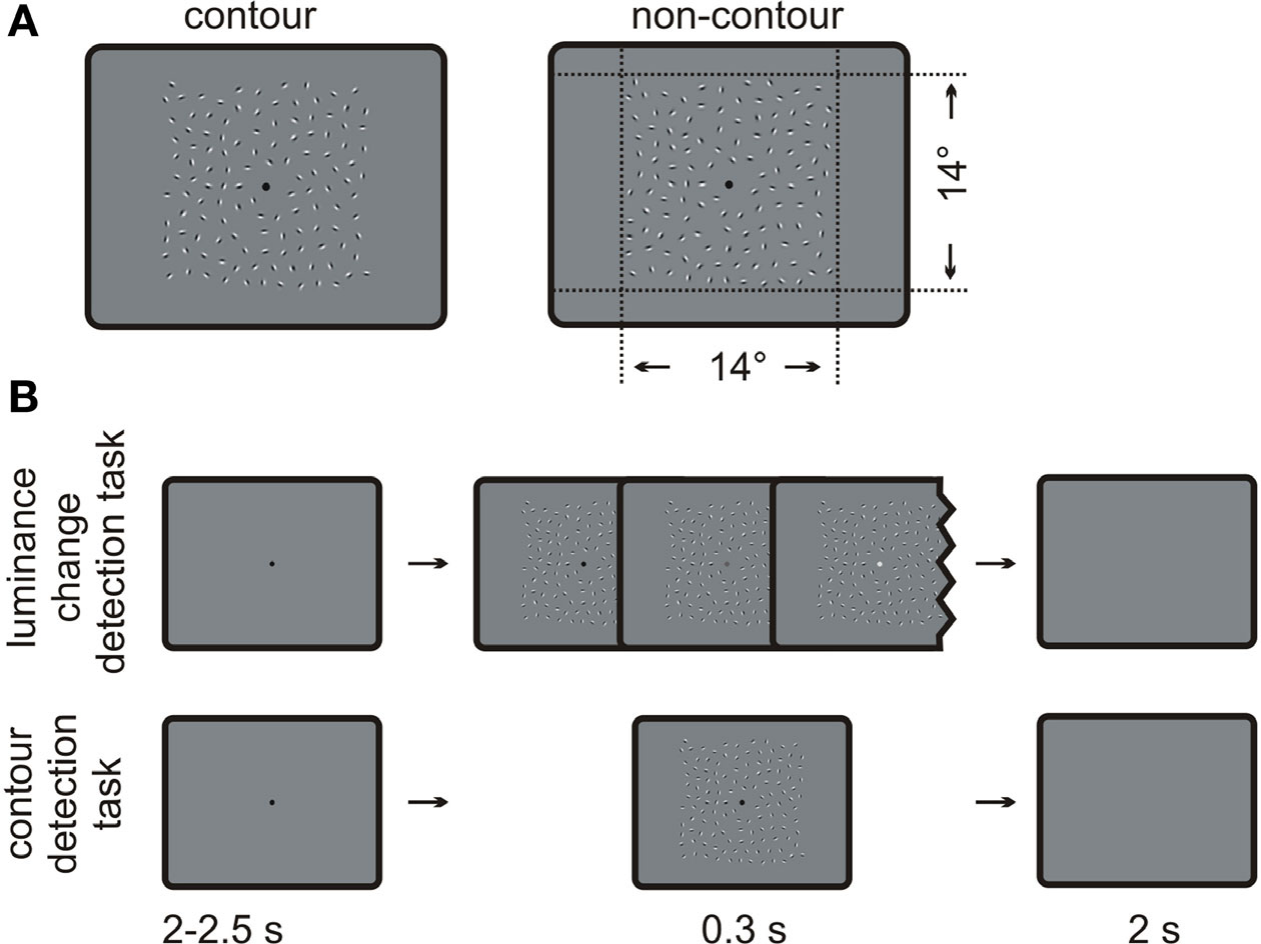
**Schematic depiction of the stimuli and the procedure**. **(A)** Examples of contour and non-contour stimuli as used in the present study. The contour stimulus on the left contains a path of nine oriented Gabor elements. **(B)** Typical trial sequences administered for the luminance change detection task (upper row) and the contour detection task (lower row).

Visual perceptual integration can be investigated with the Gabor path paradigm (Hess and Field, 1999) where observers are presented with arrays of randomly oriented Gabor patches. The arrays can contain a set of Gabor elements, orientations of which are aligned to an invisible global contour (Figure 1A), and the task is to detect the contour within contour and non-contour displays that are presented in a random order. Contour integration has been extensively examined in basic research (Mathes et al., 2006; Mathes and Fahle, 2007) as well as in clinical investigations (Del Viva et al., 2006; Silverstein et al., 2006; Butler et al., 2013; Roudaia et al., 2013). The methods include magneto- and electroencephalography (MEG/EEG, e.g., Pitts et al., 2012; Volberg et al., 2013b), functional magnetic resonance imaging (fMRI, e.g., Kourtzi et al., 2003; Altmann et al., 2004) and behavioral experiments (e.g., Hess and Dakin, 1997; May and Hess, 2007; Dakin and Baruch, 2009; Machilsen et al., 2009; Volberg, 2014). Contour integration has also been examined in non-human primates (Li et al., 2006, 2008), in cats (Crook et al., 2002; Samonds et al., 2006), and even in opossums (Oliveira et al., 2002). Taken together, there seems to be a consensus in the literature that contour integration is an essential function in the mammalian visual brain (Roelfsema, 2006; Roelfsema and Houtkamp, 2011; Wagemans et al., 2012; Shpaner et al., 2013).

Despite the large body of results, the neural mechanisms underlying contour integration are yet not fully understood. One widely investigated controversy concerns the level of processing at which contour integration occurs. A classical view is that contour grouping emerges early during visual processing, as a consequence of the neural organization of primary visual cortex V1 (Field et al., 1993). According to this view, orientation-selective neurons in V1 co-activate neurons with neighboring receptive fields and a similar orientation preference. This process results in a cascade of activations between neurons coding single segments of the Gabor path. Single-unit recordings in the cat or monkey brain indeed revealed that the presence of collinear flankers increases the neural response rates to low-contrast target Gabors in cats (Polat et al., 1998; Pooresmaeili et al., 2010), and that the facilitating effect of collinear flankers can be reduced if the cells coding the flanker orientation are inactivated (Crook et al., 2002), suggesting that lateral interactions in V1 are crucial for contour grouping. On the other hand, the results of human EEG studies suggest that contour integration is accomplished relatively late within the visual processing stream. For example, Shpaner et al. (2013) investigated differences between contour and non-contour conditions at various components of the event-related potential (ERP), including the early peaking C1 and P1 amplitudes (Di Russo et al., 2002). Contrary to what one would predict from the association field approach, the earliest differences were found during the later-peaking N1 component, 160–200 ms after stimulus onset. Whereas the actual timing of amplitude differences between contour and non-contour conditions depends on the paradigm, being delayed e.g., when using misaligned contours (Mathes et al., 2006) or a demanding task (Pitts et al., 2012), a late onset was generally found in other ERP studies (162 ms and 229 ms in Machilsen et al., 2011; ~150 ms in Mathes et al., 2006; ~215 ms in Tanskanen et al., 2008; ~ 220 ms in Mathes and Fahle, 2007; 220–260 ms in Pitts et al., 2012). Moreover, and consistent with previous fMRI studies, the neural sources on the N1 amplitude differences were located within a relatively high-tier region of the visual brain, the lateral occipital cortex (LO, Altmann et al., 2003, 2004; Kourtzi and Huberle, 2005). Shpaner et al. concluded that the LO pools information from lower tier visual regions, and that this pooling, not the lateral interaction in V1, is the initial step in contour integration.

In addition to the issue concerning the level of processing, it is still unclear whether contour integration occurs automatically. Because orientation selectivity is hard-wired in primary visual cortex, and because the activity in these neurons is fed forward into higher visual brain regions (Grill-Spector and Malach, 2004), it seems evident that contour integration emerges involuntarily and automatically (Shpaner et al., 2013). This view has been challenged as of yet in only a few studies. Li et al. (2008) presented monkeys with oriented lines embedded within background noise, and recorded the firing rates of orientation-selective V1 neurons with corresponding receptive fields. If the target line was part of a contour of collinear lines, the firing rates increased compared to a condition where the line was surrounded by randomly oriented elements. However, this facilitation critically depended on the task. Untrained monkeys that had to detect luminance changes within a central fixation dot of a contour stimulus showed no increase in firing rates, whereas a clear increase was seen when the same stimuli were presented with a contour detection task (cf. Li et al., 2006; Pitts et al., 2012). The contextual modulations of the firing rates were maintained after training after switching back to a luminance change detection task, but disappeared again if the trained monkeys were anesthetized. The results suggest that the enhancement of V1 responses to contours is not purely stimulus-driven, but is mediated by top-down processes from higher visual brain areas.

In a similar vein, we recently varied the expectancy that a contour would appear in the upcoming stimulus and investigated the consequences for contour processing (Volberg et al., 2013b). The participants were successively presented on each trial one contour stimulus and one non-contour stimulus in a random order (in a two-alternative forced-choice task). Although it was unpredictable whether the first stimulus contained a contour, the presence or absence of a contour in the second stimulus could be predicted based on the processing of the first stimulus. Contour compared to non-contour stimuli produced a marked increase in the beta-band power of EEG brain oscillations (15–19 Hz, 0–150 ms and 18–21 Hz, 170–380 ms), but only if they were presented in the first interval where the stimulus type was not predictable. Stimuli presented in the second interval produced no differences between contour and non-contour conditions. Together these results suggest that contour integration is not a purely stimulus-driven process. Rather, contour integration and the related brain differences between contour and non-contour conditions seem to require top-down processing, facilitating perception and action in the on-going task.

When viewed together, the results of the experiments addressing the level of processing and those addressing the automaticity of contour integration lead to an intriguing hypothesis that LO activity during contour processing is associated with a selection, rather than a mere passive pooling of neural information. We presume that LO is involved in top-down activity that is specific to the task of contour detection. Our hypothesis can be framed within a neuronally inspired model of perceptual grouping, the incremental grouping theory (IGT; Roelfsema, 2006; Roelfsema and Houtkamp, 2011). According to the IGT, perceptual grouping is achieved by enhancing the response of a group of neurons coding features of the same object. This is accomplished in two successive sweeps. During the feedforward sweep, networks of neurons that respond to the same feature are identified, and horizontal connections between these neurons (within the same level of the neural hierarchy) are engaged. In the subsequent feedback sweep, one element of the network is selected and its neural response is enhanced. This enhancement spreads across the network, thereby increasing the response of all connected neurons. Due to this enhancement, the group of neurons is then selected with priority over other picture elements that compete for representation (Desimone and Duncan, 1995). When proposing that contour selection is a top-down process, we refer to the idea that a feedback sweep is required in order to make the contour grouping explicit and accessible for higher brain structures. Based on the results of previous EEG and fMRI studies (e.g., Altmann et al., 2003; Shpaner et al., 2013), the lateral occipital cortex appears to be involved in this process.

Different stimulus and task factors affect the visual processing of contours (McMains and Kastner, 2011). If the resulting shape of a contour is known in advance, or if the shape corresponds to that of an already known object, then a contour can directly activate shape-selective LO neurons with large receptive fields during the feedforward sweep (“base grouping,” Roelfsema, 2006). Such a stimulus-driven grouping might especially occur for closed contours with a circular shape (Kovács and Julesz, 1993; Kourtzi et al., 2003). If the shape of the contour is unknown (i.e., if that particular shape does not correspond to the shape preference of a neuron in higher visual areas), then a mere stimulus-driven selection is not possible. In this case, we propose that contour grouping and the associated feedback from the lateral occipital cortex occurs only if the contour needs to be selected for perception and action.

In order to test this hypothesis, we adopted Li et al.'s (2008) paradigm to an EEG study with human observers. The participants were presented with contour or non-contour stimuli in a luminance-change detection task at fixation or in a contour-detection task. The contours were task-relevant only in the latter case. Moreover, the shape of the contours was random so that a stimulus-driven selection was not possible. The analyses focused on the difference in brain activity between contour and non-contour conditions. Specifically, we were interested in the neural communication between higher and lower brain areas during contour processing under different task demands. Using EEG and source-level analyses, we show that contour compared to non-contour stimuli produce increased LO activity during the N1 time range, but only if the contour is task relevant. Furthermore, we demonstrate that LO activity is associated with an inflow of information into primary visual areas in this case, consistent with the view that LO supports the grouping of contour elements.

## Methods

### Subjects

Twenty-three students of the University of Regensburg participated in the experiment. Three subjects were excluded from the analysis due to a poor behavioral performance (mean percent correct ≤65% in the contour detection task). Two further subjects had to be excluded after an initial EEG data screening, due to strong electrode artifacts. Thus, 18 participants remained in the sample (11 female, 7 male, 19–43 years, mean 22 years, standard deviation 5.5 years). They were right-handed by self-report, had no neurological disorders and normal or corrected-to-normal vision. The participants gave written informed consent prior to the experiment.

### Stimuli

Examples for contour and non-contour stimuli are given in Figure 1A.The stimulus displays contained square arrays of Gabor elements subtending 14 × 14 degrees of visual angle. Gabor elements are sine wave gratings that are multiplied with a two-dimensional Gaussian wave plane. The luminance distribution *G*(*x*, *y*) of a Gabor element can be described by Equation 1.

(1) $$G(x,y)=c\text{ cos}\left(2\pi \frac{x\text{ cos }\theta +y\;\sin\;\theta }{p}+\phi \right)exp\left(\frac{x^{2}+y^{2}}{2\sigma^{2}}\right)$$

The values *c*, *p*, θ and ϕ define properties of the sine wave grating. Value *c* is the Michelson luminance contrast, θ is the orientation, *p* is the wavelength, and ϕ is the phase of the grating. Sigma (σ) is the standard deviation of the Gaussian envelope. In the present study, the wavelength of the carrier sine wave *p* was set to 7 pixels, resulting in a spatial frequency of 2.4 cycles per degree at a viewing distance of 70 cm. The phase ϕ was π /2 so that the sine grating was odd symmetric to the center of the Gaussian envelope. The Michelson contrast *c* was set to 0.9. The background luminance as well as the average luminance of a Gabor element was 14 candela per square meter. Sigma was set to 0.12 degree, which is approximately 1/2 of the wavelength *p*.

Half of the stimuli contained a path of collinearly oriented Gabor elements, embedded within randomly oriented Gabors (contour stimulus). The strategy for generating contour stimuli was comparable to that of Watt et al. (2008). In a first step, a path consisting of nine adjacent path segments was constructed. The first path segment had a random orientation, and each following segment was tilted by plus or minus 25 ± 2 degrees relative to the orientation of the previous one. In order to avoid loops or closed contours, the solution was discarded if the difference between the first and the last segment of the path was larger than 180 degrees. One Gabor element was placed on each segment, oriented co-linear to the path. These Gabors elements made up the contour. The element separation—i.e., the distance between neighboring elements of the contour—was set to three times the wavelength *p*, corresponding to 1.2 ± 0.18 degrees of visual angle. The whole contour was copied to a random location of the stimulus array. Then, randomly oriented Gabor elements were repeatedly added to the stimulus array, with the constraint that the center of the new element was not nearer than 1.02 degrees (the minimum element separation) to the center of a neighboring element. Also, no Gabor element was placed in the screen center where a fixation dot with a 0.35 degree diameter was presented. The algorithm stopped if no more elements could be placed within the stimulus array. On average, the resulting displays contained 128 (118–138) Gabor elements.

The other half of the stimulus displays contained randomly oriented Gabor elements only (non-contour displays). They were obtained by a manipulation of the formerly constructed contour displays. Neighboring elements on the Gabor path were rotated in opposite directions by 45 degrees so that they no longer formed a continuous contour. The remaining Gabor elements were rotated by a random value. Thus, the orientation of the single Gabors elements was different but their number and the positioning was identical in contour and non-contour displays.

### Procedure

Subjects were seated in a sound-attenuated, electrically isolated chamber (Industrial Acoustics GmbH). The stimuli were presented on a 73 × 53 cm translucent screen, projected by a DLP projector located outside the chamber (NEC-V230X) that was driven with a resolution of 1280 × 1024 pixels and a vertical refresh rate of 60 Hz. A chin rest ensured that the viewing distance remained constant at 70 cm and that the head position was centered at the screen.

The experiment always started with the luminance change detection task. A typical trial sequence is depicted in Figure 1B. A black fixation dot was shown on an otherwise uniformly gray screen. After a random interval from 2000 to 2500 ms, a stimulus array was shown for 300 ms. Half of the stimuli contained a contour and the other half did not contain a contour. Moreover, on half of the trials with contour and non-contour stimuli, the brightness of the fixation dot increased linearly from black to light gray (15.9 candela/m^2^) on each 16.6 ms frame of the presentation time. The task was to indicate by button press whether the fixation dot got brighter during the stimulus presentation (yes or no, two-alternative forced choice). The participants responded with the index finger and the middle finger of one hand. The response hand as well as the mapping of fingers to the response alternatives was counter-balanced across subjects. The next trial started 2000 ms after the manual response, or 2000 ms after the presentation of the stimulus array if no response was given.

After four blocks of the luminance-discrimination task, the participants received new instructions. For the remaining blocks of trials, they were instructed to indicate whether the stimulus display contained a contour (yes or no, two-alternative forced choice). Instructions were given in a written form with example depictions of contour and non-contour arrays. At this occasion, the participants were asked whether they have seen contours as those depicted while performing the first four blocks. The trial sequence and the response buttons were identical to those applied in the luminance-discrimination task, but the fixation dot never changed luminance in the contour detection task. This was done in order to avoid the possibility that the subjects could incidentally continue to perform the luminance change detection task. The contour and non-contour stimuli were randomly drawn from a pool of stimuli and assigned to the luminance task and the contour detection task. Thus, across subjects, the contour displays did not co-vary with the two different tasks.

The participants performed four blocks with 40 trials each in the luminance-change detection task, and four blocks with 40 trials each in the contour detection task. Eighty trials in the contour detection task had to be discarded due to errors in the stimulus protocol. Thus, the subjects performed 320 trials in total, of which 240 trials were available for the analysis. Because the EEG analyses focus on differences between contours and non-contours in the luminance change detection and the contour detection tasks, the results are not affected by differences in the trial count between the tasks. Furthermore, we do not use a measure of phase concentration for which the trial count is relevant (Vinck et al., 2011).

### EEG recording and pre-processing

The EEG was recorded from 62 electrodes that were mounted in an elastic cap (EasyCap, Herrsching-Breitbrunn, Germany). Electrodes were placed on five equidistant concentric rings around Cz, where the electrodes on each ring were also spaced equidistantly. The positions on the vertical (Fpz, AFz, Fz, FCz, Cz, CPz, Pz, Iz) and horizontal (C5, C2, C1, C6) central lines were identical to those defined in the international 10% system (Oostenveld and Praamstra, 2001). For convenience, the 10% naming scheme will also be used for the remaining electrodes in the array, according to the closest matching electrode. The data were referenced to Cz during recording, and impedances were kept below 10 kOhm. The signals were amplified between 0.1 and 100 Hz and digitized at a rate of 500 Hz (BrainAmp MR plus, Gilching, Germany).

### Data analysis

The data analysis was performed on behavioral and brain responses in contour and non-contour conditions, in two different tasks where the contours were irrelevant (luminance change detection task) or relevant (contour detection task) for the behavioral response.

#### Behavioral data

Behavioral performance measures (% correct responses and reaction times in trials with correct responses) were obtained for the luminance change detection task and for the contour detection task. The sensitivity (*d*') in both tasks was also assessed. Statistics were computed with the free R language for statistical computing (R Core Team, 2012).

#### EEG data

The continuous data was segmented into epochs from −2.5 to 2.5 s, centered on the onset of the stimulus. The data were then pre-cleaned by removing epochs containing electrode or movement artifacts. In a second run, artifactual components related to eye blinks or eye movements were identified and removed from the pre-cleaned data. An infomax independent components analysis was used to that end (Delorme and Makeig, 2004). Trials with residual artifacts were finally identified and removed by visual inspection. Only trials with correct behavioral responses were included. On average, 189 trials (range 142–223 trials) per subject remained for the analysis.

The analysis strategy for the EEG data was as follows. In a first step, ERPs at the electrode level were used to identify time ranges of interest where brain activity between contour and non-contour conditions differed (method described in section ERPs). Whereas electrode-level data are useful for investigating the timing of the difference, several problems arise if these data are used for analyzing brain site communication (Schoffelen and Gross, 2009). Assessing the connectivity between each possible pair of the 62 electrodes results in (62 × 61) / 2 = 1891 single tests. A correction for multiple comparisons is then needed, which dramatically reduces the power of the analysis. Furthermore, even focal brain sources produce a broad activity on the scalp surface due to volume conduction, which is then picked up by different electrodes. Thus, connectivity between data obtained from two different electrodes does not imply that two different brain sources communicate. Finally, because the topographic distribution of brain activity can be produced by different configurations of brain sources, it remains unclear which brain sources underlie a given effect on the electrode level. These problems can be circumvented if the electrode level data are transformed into a smaller number of brain source estimates before computing the connectivity metrics (Khan et al., 2013; Larson-Prior et al., 2013). Thus, for the analyses on brain site communication we used source level data. To that end, the cortical sources of the ERP difference were reconstructed using a minimum norm technique in a second step (section Source reconstruction). In the third step, we identified connected neural sites, the activity of which co-varied with the activity of the estimated source of the ERP difference (section Connectivity analysis: power envelope correlations). To this end, source level activity was transformed into a time-frequency representation. Finally, in a fourth step directional measures of neural connectivity were used to describe the flow of information between the previously identified nodes of the brain network [section Connectivity analysis: Phase Slope Index (PSI)].

***ERPs.*** For the ERP analysis, the epochs were filtered with a zero-phase 30 Hz low-pass filter, re-sliced into epochs −200 to 800 ms relative to stimulus onset, and re-referenced to the average amplitude of two near-mastoid electrodes (TP9 and TP10). A baseline correction was performed using the whole pre-stimulus interval, −200 to 0 relative to stimulus onset.

Per subject and condition, the EEG trials were averaged to reveal the individual ERPs (contour detection task / contour stimulus, contour detection task / non-contour stimulus, luminance change detection task / contour stimulus, and luminance change detection task / non-contour stimulus). Group-level statistics were then conducted by computing paired *t*-tests between the ERP amplitudes in the contour and non-contour conditions (alpha = 0.05, two-sided). This was done for each electrode and at each sample point within a −200 to 800 ms time range relative to stimulus onset, separately for the contour detection and for the luminance change detection task. The advantage of this approach is that the time ranges or electrodes for the analysis need not be selected in advance from a priori hypotheses (Oostenveld et al., 2011). On the downside, a large number of tests must be conducted so that a correction for multiple comparisons is necessary. To correct for multiple comparisons across electrodes, a permutation approach was used (Blair and Karniski, 1993). At each time point of the baseline interval (−200 to 0 ms), 1000 *t* tests were performed where the contour and non-contour conditions were randomly swapped within participants. The number of electrodes showing significant differences between contour and non-contour conditions was recorded after each run. Using data from the baseline interval for the permutation has the advantage that the same null distribution is used to assess differences at each post-stimulus sample point, which is not the case in a sample point-wise permutation procedure (see Volberg et al., 2013a, for a previous application; cf. Maris and Oostenveld, 2007). This procedure generates a distribution for the number of significant electrodes that can be expected under the null hypothesis assuming no difference between the contour and non-contour conditions. The results of the first stage analysis were only accepted if the number of significant electrodes, at a given time point, exceeded the 0.95 quantile of the null distribution. With this procedure a correction for multiple comparisons across electrodes was achieved. To also correct for multiple comparisons across sample points, we only considered differences that were significant in six or more consecutive sample points (i.e., 12 ms or longer). This strategy has been successfully applied in several previous publications by our group (e.g., Hanslmayr et al., 2011; Volberg et al., 2013b).

***Source reconstruction.*** The cortical source activity was reconstructed at time ranges where significant differences between ERPs in the contour and in the non-contour condition were observed. The forward model for the source reconstruction was computed using the OpenMEEG plugin for fieldtrip software (Kybic et al., 2005; Gramfort et al., 2010). A forward model relates source level activity to activity at the electrode level. The quality of the model strongly depends on two factors: An accurate description of the individual head anatomy, and a precise measure of the individual electrode locations on the scalp.

In order to reveal high-quality forward models, individual head shapes were obtained from structural magnetic resonance imaging with a 3-T MR head scanner (Siemens Allegra, Erlangen, Germany). For each participant we acquired high-resolution sagittal T1-weighted images using a magnetization prepared rapid gradient-echo sequence (MP-RAGE; repetition time = 2250 ms; echo time = 2.6 ms; 1 mm isotropic voxel size). Realistic three-layer boundary element head models were then constructed for the tissues brain, skull, and skin. The relative conductivity of the tissues was set to 1, 1/30, and 1, respectively. Source positions were placed on a regular 5-mm grid covering the whole brain compartment. The actual number of source positions depended on the individual brain anatomies and ranged between 9037 and 15866 across participants. To further improve the quality of the forward model, the electrode locations were measured individually after attaching the electrode cap and prior to the actual EEG recording. An ultrasonic-based 3D digitizer (ELPOS, Zebris, 88316 Isny, Germany) was used to that end. The electrode positions were determined relative to three anatomical landmarks (nasion, left pre-auricular point, right pre-auricular point) and then transformed into individual head coordinates by aligning the three fiducials with the same landmarks in the structural MR scans. The resulting forward models were based on individual head anatomies as well as on individually measured electrode locations, and as such approached the highest achievable quality.

As an inverse solution, minimum norm estimates were computed (MNE, Hämäläinen and Ilmoniemi, 1994). The inverse solution relates electrode level data to the activity of cortical sources as specified in the forward model. The source activity in the contour and the non-contour conditions were then compared by means of paired *t*-tests at all source positions simultaneously. Analogous to statistical parametric mapping of functional magnetic resonance imaging data (SPM, www.fil.ion.ucl.ac.uk/spm), the resulting volumetric source data represents a statistical map of source activity differences between the contrasted conditions. The map was interpolated on the individual brain anatomy (voxel size 1 × 1 × 1 mm) and projected onto an inflated standard brain provided with Caret software (brainmap.wustl.edu/caret.html; van Essen et al., 2001). Similar to a strategy that is often used in whole-brain analyses of brain imaging data (e.g., Hanslmayr et al., 2013), we considered only source differences that were significant in a minimum number of neighboring voxels. After an initial data inspection, the threshold was set to 1000 voxels, corresponding to a brain patch of one cubic centimeter. The thresholding revealed a small number of physiologically plausible sources, suggesting that the threshold was chosen adequately. Anatomical labels were derived from the Talairach and Tournoux (1988) stereotaxic atlas.

***Connectivity analysis: power envelope correlations.*** Functional connectivity was investigated by means of power envelope correlations within source level activity (Hipp et al., 2012). A power envelope connects the peaks of the amplitude of an oscillation. Notably, the power envelope does not co-vary with the phase. Thus, two oscillations with co-varying amplitudes but different phases will have positively correlated power envelopes. The power envelope correlation method requires that, for each source location of interest, a time-domain signal is reconstructed and decomposed into its spectral components. To that end, a linearly constrained minimum variance beamformer inverse solution (LCMV; Sekihara et al., 2004) was computed on the same forward models that were used for the MNE solution. The LCMV method reveals spatial filters that can be used to compute single trial source activity from the EEG channel data. The source signals were then decomposed into time-varying spectral components by convolving a 7-cycle sinusoid at a given frequency with a data segment of the same length. Prior to the convolution, the data segment was multiplied with a Hanning window. The data were filtered every 10 ms, in steps of 1 Hz from 4 to 40 Hz. Event-related power changes were investigated by computing the percent signal change relative to an average baseline activity. The baseline comprised 500 ms prior to the stimulus onset. The baseline for time-frequency analyses should contain several cycles of the slowest investigated oscillation (4 Hz in our case) and was therefore larger than the baseline for the ERP analysis.

The power envelope correlation method allows us to identify brain regions that are connected to a given seed region. Because we were interested in brain sites underlying contour processing, the neural source of the ERP difference between contour and non-contour trials as obtained from the MNE solution served as a seed region in this study. ERP differences were observed only during blocks where the participants were required to detect contours, so that the source connectivity analysis could be restricted to this task. All trials presented in the contour detection task were entered into the analysis. The single-trial source signal at the seed region was reconstructed using spatial filters and was decomposed into a time and frequency representation. In order to account for individual variability of the neural source activity, a 1 cm sphere was constructed around the source location where the grand mean difference between contour and non-contour conditions was maximal. For each individual, the voxel showing the largest difference within that sphere was then selected as the seed region for further processing (see Hanslmayr et al., 2013). Signal components with the same phase (reflecting activity of the same source) were removed before computing the power correlation.

Prior to computing the actual correlations it is necessary to select frequencies of interest on which the power estimates are performed. Based on previous findings, we focused on beta frequencies (~15 Hz) where we expected larger power in the contour compared to non-contour condition (Volberg et al., 2013b). In order to validate this selection, two analyses were performed. First, power differences in the source level activity were identified by applying paired *t*-tests (alpha = 0.05) on the mean beta power in contour and non-contour conditions at each time and frequency bin. Suitable beta frequencies were identified by visual inspection of the time-frequency representation in the contour vs. non-contour condition. Second, a control analysis was performed on the electrode level. Using the previously identified time and frequency ranges, head topographies on the beta power difference between contour and non-contour conditions were constructed. According to the results of Volberg et al. (2013b) we expected differences in occipital and occipito-parietal electrodes. For the statistical analysis, the data was thus averaged over occipito-parietal electrodes and subjected to paired *t*-tests (contour vs. non-contour, alpha = 0.05). Because the hypotheses were directional, one-sided tests were used in each case.

The statistical analysis was similar to that applied in the original work by Hipp et al. (2012). For each subject, the power envelope correlations were computed between the estimated source activity at the seed region in each single trial and the estimated source activity at other source locations within the same trial. This was done for each source position on the individual 5 mm brain grid (see section Source reconstruction). Then, across subjects, one-sample *t*-tests were applied in order to test whether the correlation between the power envelopes at a given source location and the seed region was larger than the grand mean average correlation between the power envelope of all source positions with the power envelope at the seed region. The individual volumetric data was interpolated onto the MNI (Montreal Neurological Institute, www.mni.mcgill.ca) template brain distributed with the SPM8 toolbox for MATLAB to that end (www.fil.ion.ucl.ac.uk/spm). Because correlations are not normally distributed, they were transformed to Fisher's z correlations prior to the statistical analysis. For the data presentation, the average Fisher's z correlation at significant source positions was back-transformed into a Pearson correlation. As for the ERP source reconstruction (section Source reconstruction), only those power correlations are reported that were significant in 1000 or more neighboring voxels. This inclusion criterion helps to reduce the number of nodes for the subsequent connectivity analysis.

***Connectivity analysis: Phase Slope Index (PSI).*** Power envelope correlations reveal connectivity patterns across the whole-brain. The technique is suitable for identifying nodes within a brain network. It does not reveal, however, directional measures of information flow. We used the phase slope index (PSI, Nolte and Müller, 2010) as a measure of directional information flow. The method distinguishes a driver and a recipient in bivariate time-series data by quantifying the relative delay between signals. If series *i* precedes (or drives) time series *j*, then the phase difference of an oscillation increases with increasing frequency so that the slope of the phase difference over the frequency is positive. Likewise, a negative index indicates that time series *j* precedes (drives) times series *i*.

More formally, the PSI is based on the slope of phase differences between two time series *i* and *j* over frequencies *f*. If the time series are merely delayed by a time τ, then the phase difference would increase linearly with the frequency, proportional to τ. This behavior can be quantified from the coherency *C*_*ij*_, which is a normalized version of the cross spectrum between the time series *i* and *j*. The PSI is defined as ${\tilde{\Psi }}_{ij}=\;ℑ\left({\sum_{f\in F}C^{*}}_{ij}\left(f\right)\;C_{ij}(f+\delta f)\right)$, where *i* and *j* are two time series, *f* is the frequency, δ *f* is the frequency resolution, and ℑ〈·〉 denotes the imaginary part of the complex-valued quantity in 〈 · 〉. A crucial parameter in the PSI analysis is *F*, which indicates the set of frequencies (the bandwidth) over which the phase slope is summed. This parameter will be specified in the results section, based on the outcome of the power envelope correlation. As a final step, ${\tilde{\Psi }}_{ij}$ can be normalized by an estimate of the standard deviation, $\Psi_{ij}={\tilde{\Psi }}_{ij}\;/\;std({\tilde{\Psi }}_{ij})$. The normalized quantity was used in the present manuscript. PSI analyses were conducted between the seed region and all nodes that were identified by power envelope correlations. The analyses were done separately for contour and for non-contour conditions. With a given bandwidth, the PSI analysis reveals a spectrum of PSI values, similar to a time-frequency representation. Because the relevant time points and frequencies were unknown a priori, the PSI analysis was conducted over the whole −0.5 to 1 s data segment and between 1 and 40 Hz, in steps of 10 ms and 1 Hz.

For the statistical analysis, paired *t*-tests were conducted between the PSI value in contour and non-contour conditions, at each time and frequency bin. A permutation procedure was conducted in order to correct for multiple comparisons. In 1000 successive runs, the data obtained in contour and non-contour conditions were randomly swapped within subjects. The largest number of significant *t*-tests that were obtained in adjacent time-and-frequency bins was recorded. For example, if a difference was significant from 10 to 12 Hz and from 250 to 270 ms, then the effect would cover 3 by 3 = 9 adjacent bins, given a frequency resolution of 1 Hz and a time resolution of 10 ms. Similar to the approach applied to the ERP data, data from the pre-stimulus baseline interval (−500 to 0 ms) were used for the permutation, in order to reveal a common null distribution for statistical testing. A PSI difference was only considered significant if the number of adjacent significant time-frequency bins in the actual data exceeded the 0.95 quantile of the permutation distribution.

***Individual differences and behavioral correlates.*** For those differences between contour and non-contour conditions that were significant in the group level analysis, we finally investigated how consistent this difference was across the individual subjects. Because a consistency metric is difficult to achieve for topographic and volumetric data (ERP head topography, ERP source reconstruction, source power envelope correlations), the analyses focused on differences obtained from specific electrodes or specific source locations. Per subject, ERP amplitude differences, source power differences at the seed region for the connectivity analysis, and PSI differences between the seed regions and possible target regions were obtained. Further selections of electrodes, time ranges or frequencies depend on the outcome of the previous analyses and are reported in the results section. For each measure, the number of subjects showing a difference consistent with the group level result was computed. Binomial test were then applied in order to test whether the number is larger than that which can be expected by chance (with *p*_0_ = 0.5, *n* = 18, alpha = 0.05). Finally, for each measure, the difference in the contour minus non-contour conditions was correlated with the available data on the behavioral performance, i.e., reaction times, hit rates, and *d*' measures (Pearson correlation, alpha = 0.05, two-sided).

## Results

### Behavioral results

Subjects responded correct in 98.85 ± 1.07% (mean ± standard deviation) of trials in the luminance change detection task and in 82.97 ± 7.74% in the contour detection task on average. The corresponding sensitivity indices *d*' were 5.23 ± 0.73 and 2.17 ± 0.87, respectively. Reaction times were 742 ± 135 ms in the luminance change detection task and 853 ± 161 ms in the contour detection task. Only one out of 18 participants reported that she saw contours while performing the luminance change detection task. Compared to the other subjects, this participant also showed a lower response accuracy and a marginally higher response latency in the luminance change detection task [96.25% correct, *t*_(16)_ = 8.58, *p* < 0.001 and 818 ms, *t*_(16)_ = −2.12, *p* = 0.05 by one-sample *t*-tests].

### EEG results

#### ERPs

**Figure 2:**
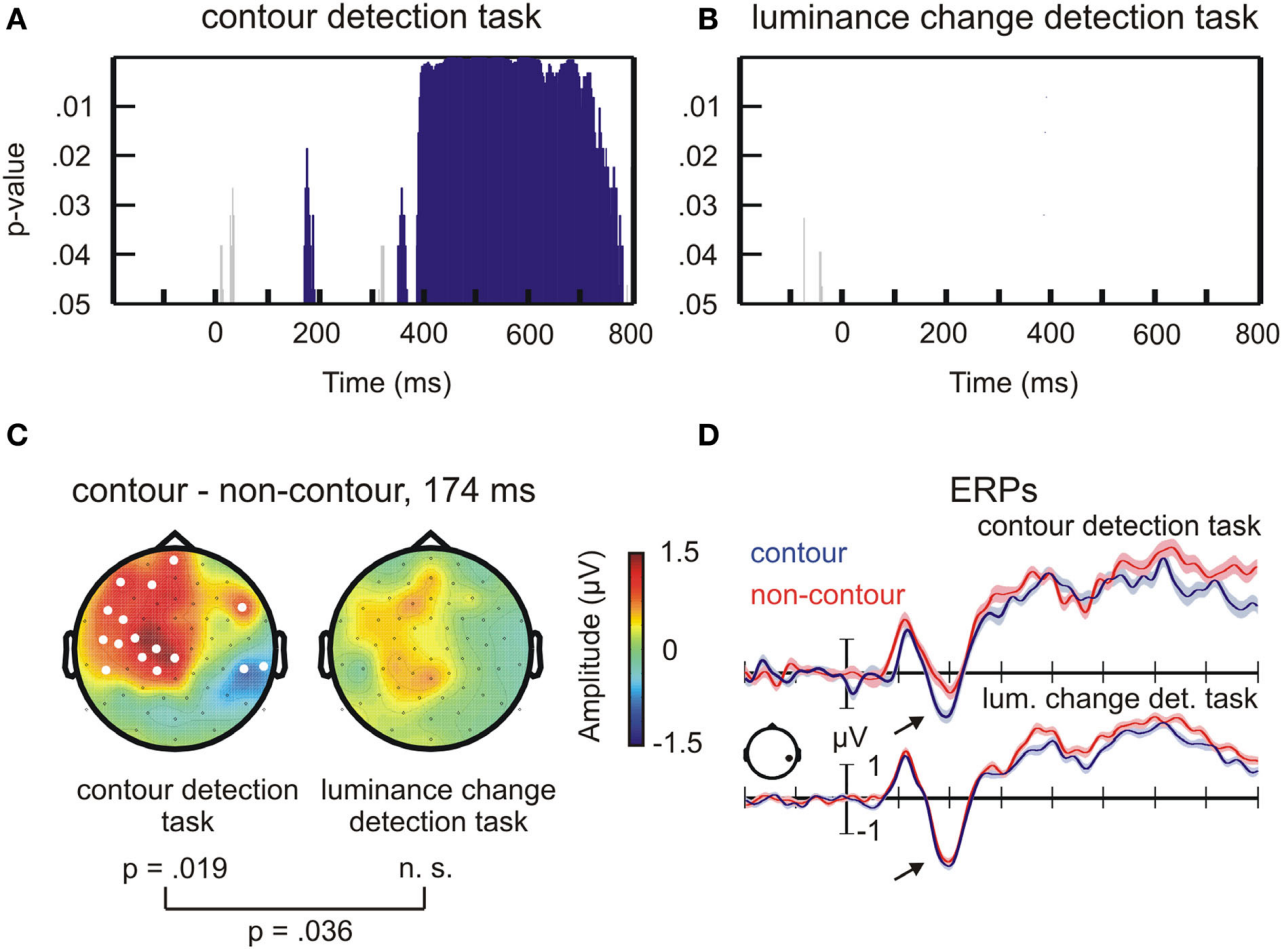
**Results of the permutation test on ERP amplitude differences between contour and non-contour stimuli**. **(A)** Statistical results for the contour detection task. The blue bars mark time ranges where the difference between contour and non-contour conditions was significant by permutation test. Short-lasting differences were not further considered (gray bars, see text for details). **(B)** Same as **(A)**, but for the luminance change detection task. **(C)** Head topographies of ERP difference waves, contour minus non-contour conditions, at the N1 peak difference. **(D)** ERP waveforms for contour and non-contour conditions at representative electrode P6 (mean ± standard error of the mean for repeated measures).

The results of the sample-wise comparison between contour and non-contour conditions are given in Figures 2A,B. Depicted is the *p*-value for the comparison, after correction for multiple comparisons as described in section ERPs.

For the contour detection task, significant differences were observed between contour and non-contour conditions from 168 to 188 ms, from 348 to 366 ms, and from 384 to 782 ms after stimulus onset (Figure 2A). Within these time ranges, the number of significant electrodes exceeded the critical number of electrodes obtained by permutation tests (critical *n* = 11 electrodes). Corresponding differences between contour and non-contour conditions did not occur in the luminance detection task (Figure 2B; critical *n* = 12 electrodes). Only four sample points showed significant differences in this task. All of them occurred during the pre-stimulus baseline interval.

Most interesting for the present purpose was the difference between 168 and 188 ms for contour vs. non-contours in the contour detection task. The maximum number of significant electrodes was reached after 174 ms where 16 electrodes were involved (*p* = 0.019 by randomization test; Figure 2C, left). At this time point, the effect had bipolar topography showing more positive amplitudes for contour compared to non-contour conditions at left centro-frontal electrodes, and more negative amplitudes at right parietal and temporo-occipital electrodes. The differences between contour and non-contour conditions in the luminance detection task showed a similar topography, but were less pronounced and generally not statistically significant (Figure 2C, right). To test for an interaction between the stimulus type and the task, a permutation procedure was applied revealing the difference between the number of significant electrodes in the contour detection task and in the luminance change detection task that can occur by chance, at the 174 ms time point where the largest difference between contour and non-contour conditions was observed. The critical value was *n* = 14 electrodes. Thus, the actual difference in 16 electrodes was significant according to the randomization test (*p* < 0.036).

Compared to the results of previous studies, the topography for the contour detection task in Figure 2C shows a more pronounced frontal difference and a weaker parietal difference between contour and non-contour conditions. This can be explained by the fact that we used electrodes TP9 and TP10 as a reference. The average of these electrodes is subtracted from all other electrodes during re-referencing, thereby cancelling out amplitude differences at nearby parietal electrodes. When transforming to average reference, the topography shows a pattern with a broad parietal negativity for contour minus non-contour conditions (Supplemental Figure 1). Thus, the result for the contour detection task closely matches those obtained in previous studies (e.g., Shpaner et al., 2013).

The ERPs are shown for one posterior electrode where the difference between contour and non-contour conditions was significant (P6, Figure 2D; significant electrodes are marked in white in Figure 2C). A more marked negativity (arrows point to this difference in Figure 2D) for contours compared to non-contours is visible in the N1 amplitude. This difference, however, only occurred in the contour detection task. The shaded area mark ± one standard error of the mean, obtained with the normalization method for repeated measures designs (Franz and Loftus, 2012). Although the trial number per condition was relatively low, the shown ERPs had a good signal-to-noise ratio (small error margins) due to the relatively large subject sample size.

#### ERP sources

**Figure 3:**
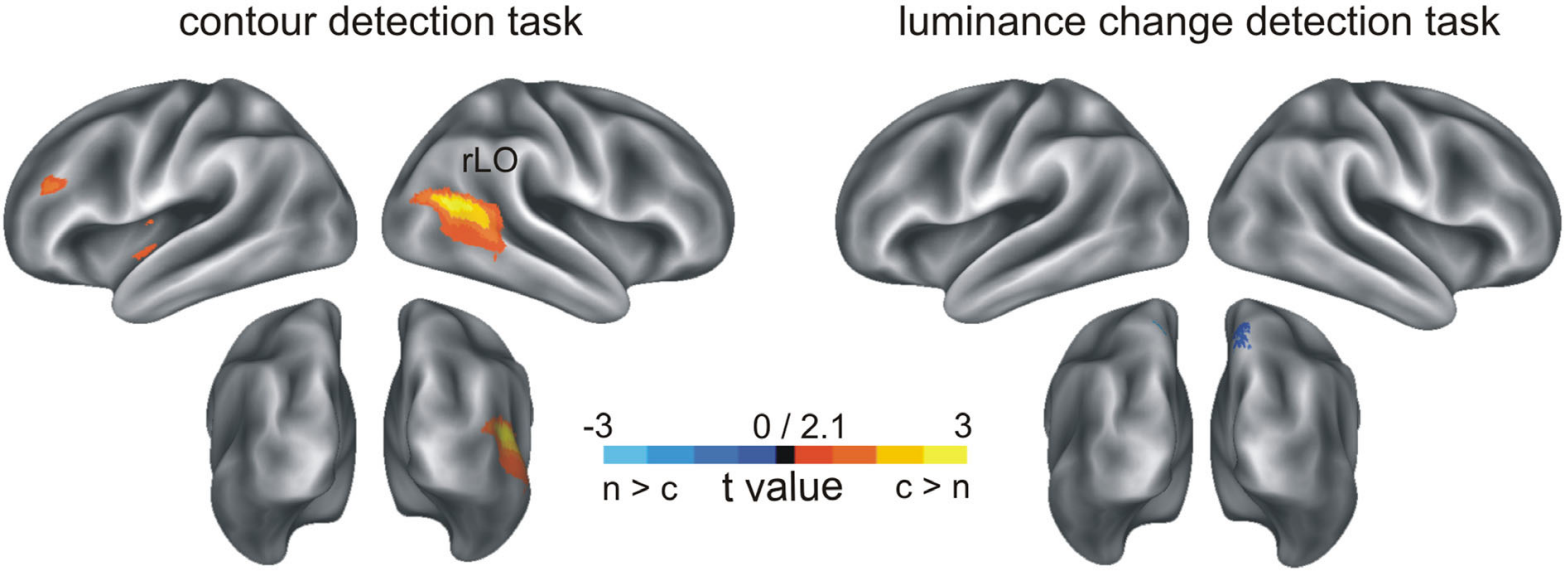
**Results of the MNE inverse solution on ERP N1 amplitude differences**. Depicted are t-maps for source activity differences between contour and non-contour conditions. Blue color indicates a larger source activity in the non-contour compared to the contour condition, and red-to-yellow color indicates larger activity in response to contour compared to non-contour stimuli.

In a next step, the neural sources of the ERP difference between contour and non-contour conditions were reconstructed, within the 168–188 ms time range of interest as obtained from the ERP analysis. The results (*t*-maps) are summarized in Figure 3 and in Table 1.

**Table 1:** **Results of the MNE inverse solution on ERP N1 amplitude differences (see Figure 3)**.

	***k***	**MNI (x, y, z)**	**Anatomical label**
**CONTOUR DETECTION TASK**			
**c > n**	23752	30, −65, 11	middle occipital gyrus, temporal gyrus, superior temporal gyrus
	2290	−33, 5, 1	claustrum, insula
	1172	−43, 34, 34	middle frontal gyrus, superior temporal gyrus
**n > c**	–		
**LUMINANCE CHANGE DETECTION TASK**			
**c > n**	–		
**n > c**	1171	10, −68, 69	precuneus

For each source the following is indicated: the number of connected significant voxels k, the MNI coordinates of the largest t value within the cluster, and the anatomical labels according to the Talairach and Tournoux (1988) atlas. (c, contour; n, non-contour).

Consistent with the results of the ERP analysis, in the luminance-change detection task the source reconstruction revealed generally no indication of increased brain activity in the contour compared to the non-contour condition (largest number of clustered significant voxels *k* = 86). However, there was a small effect in the precuneus (~BA7, MNI coordinates *x* = 10, *y* = −68, *z* = 69; cluster size *k* = 1171) where the estimated brain activity was larger in the non-contour compared to the contour condition.

In contrast, for the contour detection task there were three clusters, all of which showed larger brain activity estimates in contour compared to non-contour conditions. By far the largest cluster occurred in the right lateral occipital cortex (rLO, *k* = 23752). The estimated activity covered the middle occipital as well as the middle temporal and superior temporal gyrus (~BA37). The maximum was seen at the MNI coordinates *x* = 30, *y* = −65, *z* = 11. Two further clusters were reconstructed in more frontal regions. One occurred within the left lateral sulcus, covering the claustrum as well as the insula (*k* = 2290). The maximal *t*-value was observed at MNI coordinates *x* = −33, *y* = −5, *z* = 1. The other frontal cluster occurred in the left middle and superior frontal gyrus, (~BA9, *x* = −43, *y* = 34, *z* = 34; *k* = 1172).

#### Source connectivity

In accordance with previous research, the source reconstruction revealed that the major source of the N1 ERP amplitude difference was in the lateral occipital region, rLO. Therefore, rLO was selected as the reference site for the power envelope correlation analysis (MNI coordinates *x* = 30, *y* = −65, *z* = 11).

**Figure 4:**
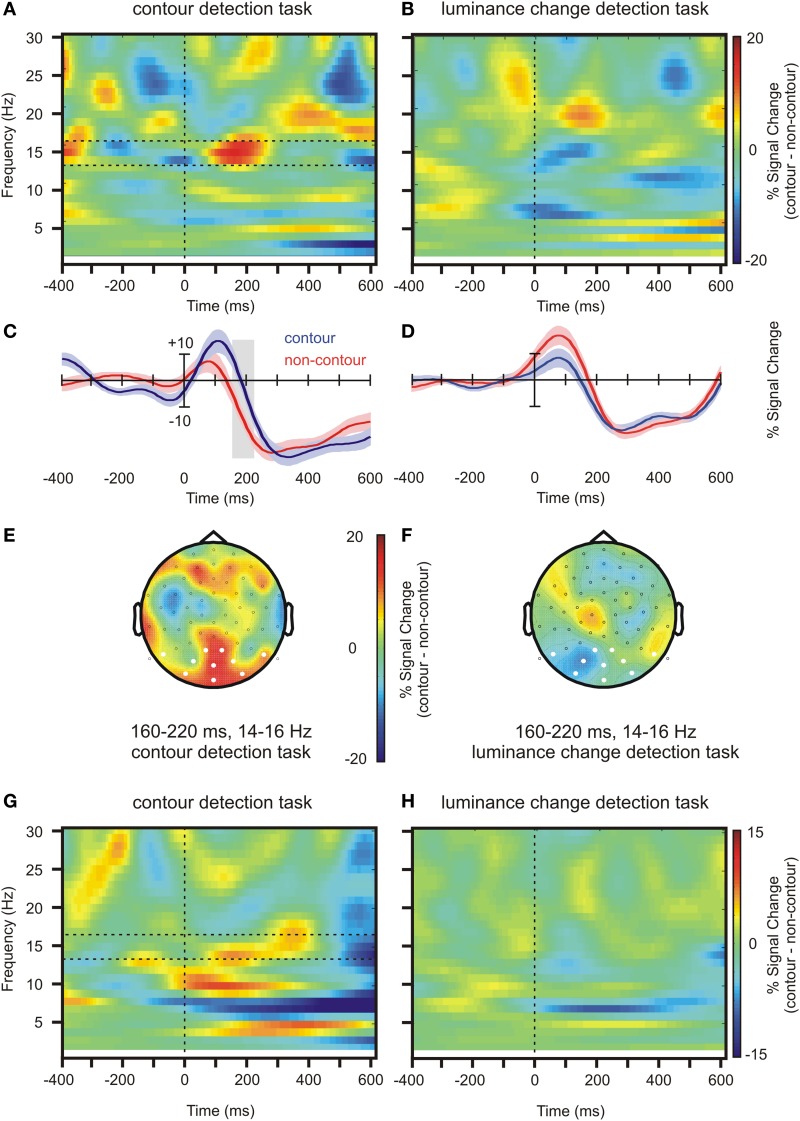
**Oscillatory activity at the source level (seed region rLO, see Figure 3) and at the electrode level**. **(A)** Time and frequency representation of the source activity, contour minus non-contour condition, in the contour detection task. The horizontal dashed lines show the range of frequencies that was selected for the further analyses **(B)** Same as **(A)**, but for the luminance detection task. **(C)** Waveforms showing the average beta power (14–16 Hz) in contour and non-contour conditions, contour detection task. The shaded areas mark ± 1 standard error of the mean for repeated measures designs. **(D)** Same as **(C)**, but for the luminance change detection task. **(E)** Head topography showing beta power differences in contour minus non-contour conditions at the electrode level (14–16 Hz, contour detection task). **(F)** Same as **(E)**, but for luminance change detection task. **(G,H)** Same as **(A,B)**, but for electrode level activity. The figures show the average activity at posterior electrodes as marked in white in **(E)** and **(F)**.

Time-domain source signals were reconstructed by means of spatial filters as obtained from an LCMV inverse solution. The filters were constructed from the covariance matrix of the channel ERP data, within the peak of the N1 component (168–188 ms) where the ERP differences were significant. In order to reveal a suitable frequency range for the power envelope correlation, the source signal at the seed region was decomposed into time-varying spectral components and the time-frequency representations in the contour and in the non-contour conditions were compared. The results are depicted in Figure 4. They show grand mean average power differences between contour and non-contour conditions, separately for the contour-detection task (Figure 4A) and for the luminance-change detection task (Figure 4B). Of special interest were power modulations in latency range of the N1 component (~180 ms), where significant results were obtained in the ERP analysis and in the related source reconstruction. As expected, inspection of the data revealed a prominent increase in beta power (14–16 Hz) for contour compared to non-contour conditions during the contour detection task. Both stimuli produced a beta power increase followed by common decrease, where the beta power was larger in the contour compared to the non-contour condition (Figure 4C). The difference was significant in seven consecutive time bins between 160 and 220 ms (alpha = 0.05, one-sided), thus exceeding our criterion for significance as defined in section II.5.2.1. The maximal difference was reached at 170 ms, after the peak and during the common decrease of beta power. Corresponding differences did not occur in luminance detection task (Figures 4B,D). The interaction between stimulus type and task (14–16 Hz, 160–220 ms) was significant, *F*_(1, 17)_ = 4.52, *p* = 0.05.

Figures 4E,F show beta power differences between contour and non-contour conditions on the electrode level in the same time and frequency range, separately for the contour detection task and the luminance change detection task. Similar to the results reported in Volberg et al. (2013b), in the contour detection task contour stimuli produced a larger beta power than non-contour stimuli at posterior electrodes. A corresponding difference was not observed in the luminance change detection task. The power in the contour and non-contour conditions was averaged over the posterior electrodes as marked in white in Figures 4E,F, and subjected to paired *t*-test separately for the contour detection task and the luminance change detection task (alpha = 0.05, one-sided). The difference was significant in the contour detection task, *t*_(17)_ = 1.88, *p* = 0.039, but not in the luminance change detection task, *t*_(17)_ = −1.59, *p* > 0.9. Also, the interaction between task and stimulus type was significant, *F*_(1, 17)_ = 6.82, *p* = 0.018. Figures 4G,H show the corresponding time-frequency representations of the beta power in the contour minus non-contour conditions, averaged over posterior electrodes as described above. Although the beta power differences occurred at somewhat lower frequencies compared to Volberg et al. (2013b), in the contour detection task they showed a similar pattern with an earlier difference in lower beta frequencies (~13–15 Hz) and a subsequent difference at higher beta frequencies (~15–18 Hz). No such difference occurred between contour and non-contour conditions in the luminance change detection task.

**Figure 5:**
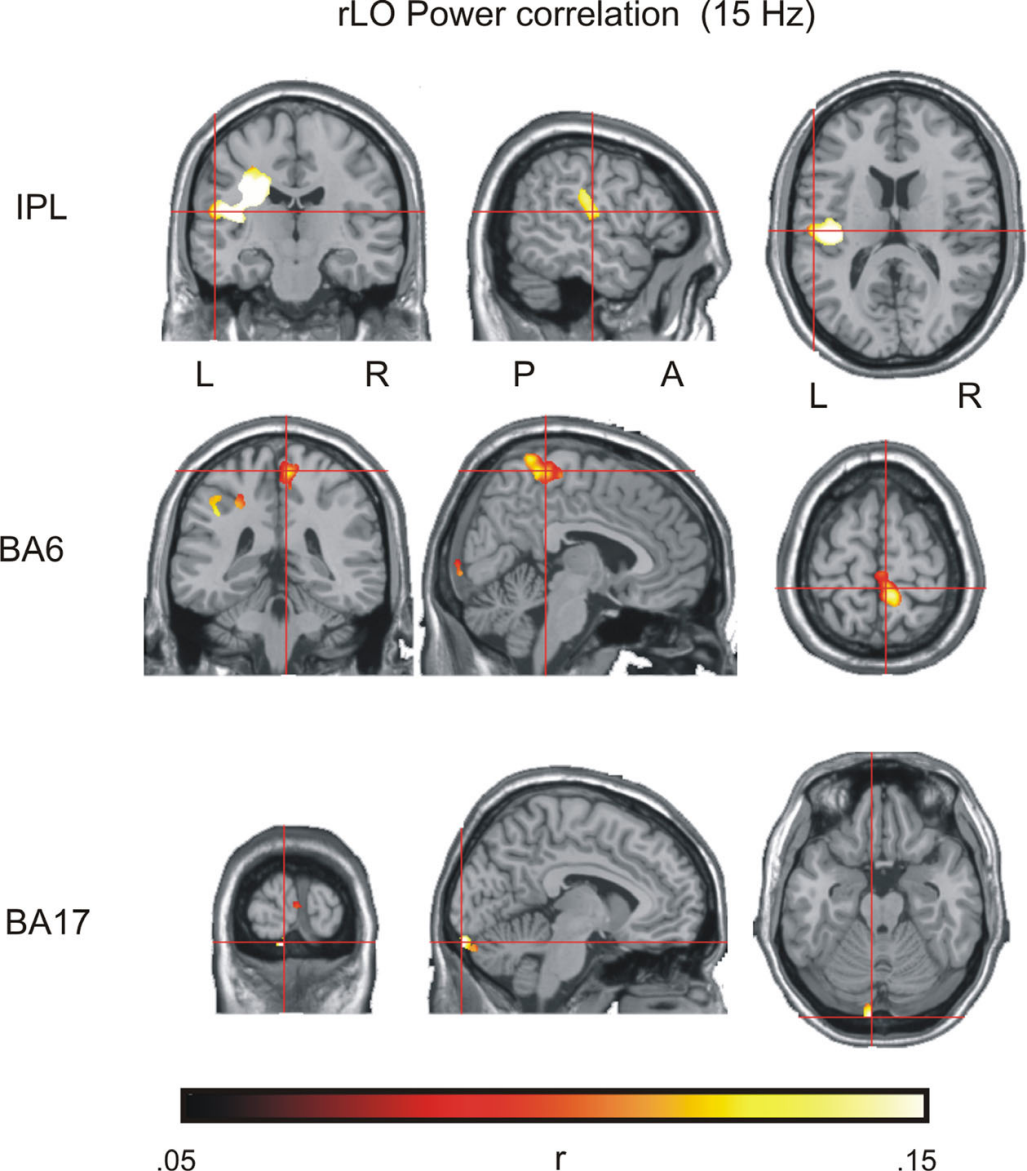
**Results of the power envelope correlation in the contour detection task, with rLO as a seed region (see Table 2 for details)**.

The results of the source power analysis were used to restrict the time and frequency range for the subsequent power envelope correlation analysis. A 15 Hz target frequency was chosen where the difference between contour and non-contour conditions was most pronounced. The 160–220 ms time range identified in the statistical analysis would not allow for a reasonable frequency resolution. Therefore, a time range between 120 and 220 ms was used. This time range is centered on the maximum of the source beta power difference at 170 ms. The previously identified source of the ERP differences between contour and non-contour trials, rLO, served as a seed region. The results are shown in Figure 5 and Table 2. Following the procedure suggested by Hipp et al. (2012), only those correlations are shown that were significantly larger than the average correlation coefficient of all source positions with the seed region. As for the ERP source analysis, we restrict this further analysis to sources that comprised 1000 or more voxels in the interpolated source space. For the interpretation of the results, please mind that Figure 5 depicts residual correlations between the power envelopes of orthogonalized signals (frequency components with the same phase removed), which have a much lower magnitude than correlations between non-orthogonalized signals (Hipp et al., 2012).

**Table 2:** **Results of the power envelope correlation in the contour detection task (15 Hz, 120–220 ms), with rLO as a seed region (see Figure 4)**.

***k***	**MNI (x, y, z)**	**Anatomical label**
14401	−53, −18, 15	insula, postcentral gyrus, inferior parietal lobe
3905	6, −37, 66	BA 6, medial frontal gyrus, paracentral lobule
1132^*^	−6, −98, −21	BA 17, BA 18, lingual gyrus

For each source is indicated: The number of connected significant voxels k, the MNI coordinates of the largest correlation r within the cluster, and the anatomical labels according to the Talairach and Tournoux (1988) atlas.

* summed over eight neighboring clusters within the occipital cortex.

There were three large clusters within the beta network as estimated from the EEG. The largest cluster comprised 14401 voxels covering the posterior parietal lobe and the postcentral gyrus. The maximum was found in the inferior parietal lobe (IPL; MNI coordinates *x* = −53, *y* = −18, *z* = 15). A second cluster was found over medial frontal and paracentral sites. It comprised 3905 voxels and had a maximum in BA6 (MNI coordinates *x* = 6, *y* = −37, *z* = 66).

Striking correlations were also found with source estimates in occipital regions where eight different clusters showed up. The clusters were not contiguous, but occurred in a circumscribed area of the visual cortex within the cuneus, lingual gyrus and BA17/18, with a maximum activity in BA17 (MNI *x* = −6, *y* = −98, *z* = −21). Together the eight clusters comprised 1132 significant voxels. Given that the combined activity within the occipital cortex exceeded our 1000 voxel threshold, we included the occipital source into the final source model.

The analysis also revealed a fourth cluster in right BA37 (MNI: *x* = 55, *y* = −69, *z* = −14). This cluster spatially overlapped with the seed region and likely reflects activity within the same neural source. It was therefore not included as a separate source into the further analysis.

#### Phase slope index

The PSI was computed for the three possible pairings of the nodes of the identified beta network (IPL, BA17, BA6) and the seed region rLO (i.e., BA17—rLO, BA6—rLO, and IPL—rLO). Since the seed region and the connected nodes were only identified for the contour detection task, the initial set of PSI analyses was also restricted to these data. A bandwidth of 5 Hz was chosen for the PSI computation, which corresponds approximately to the frequency range where the beta power difference was observed at the seed region (see Figure 4).

**Figure 6:**
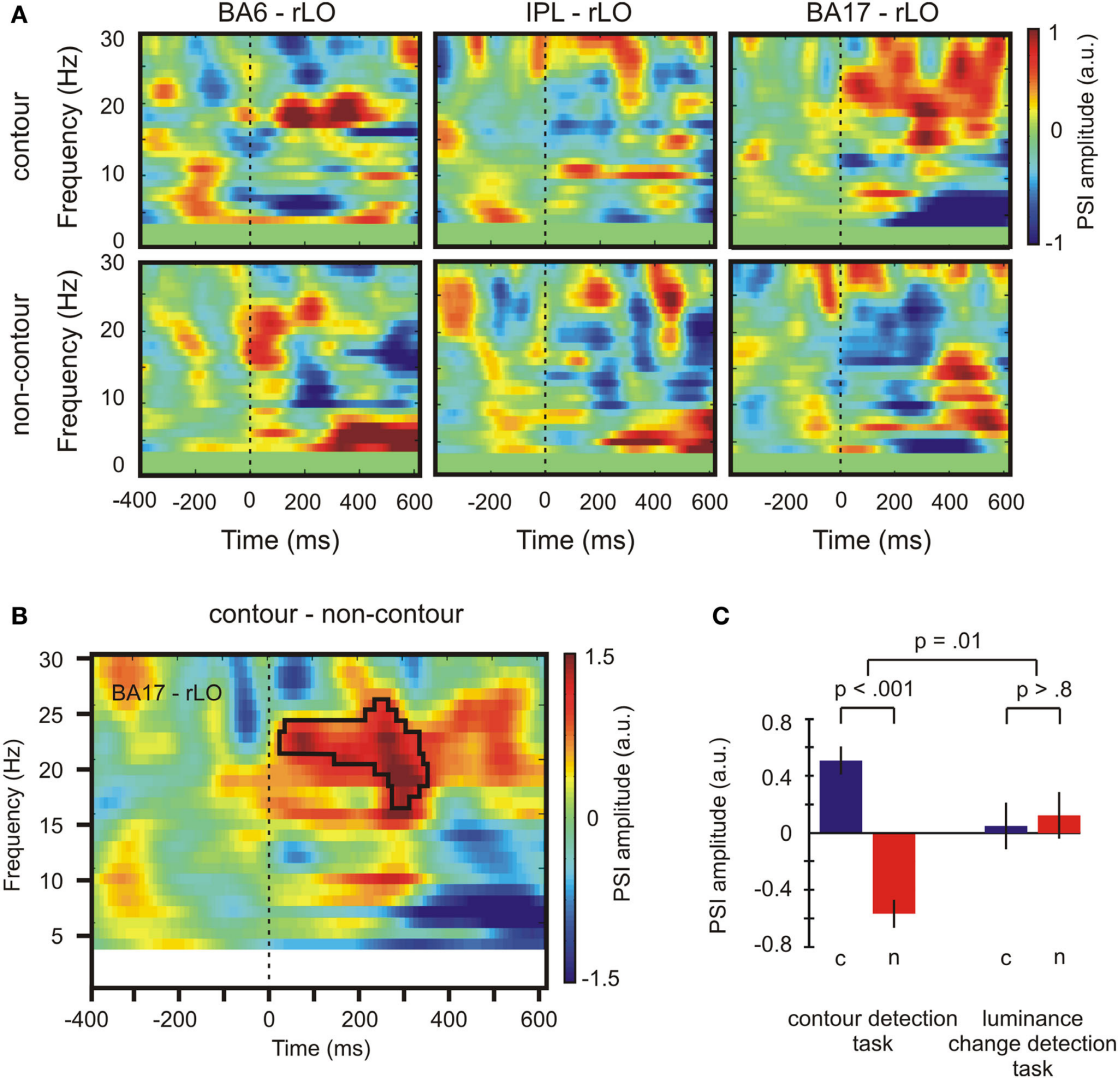
**Results depicting the directional information flow between primary visual and lateral occipital areas. (A)** PSI values for contour and non-contour conditions during the contour detection task. **(B)** Time-varying PSI spectrum for source-domain time series from BA 17 and rLO. The black frame marks time and frequency bins where the difference between contour and non-contour PSI was significant by permutation test. **(C)** Average PSI amplitude for contour (c) and non-contour (n) trials in both tasks for significant time and frequency bins as marked by the black frame in **(B)**. Positive values indicate that rLO drives BA17 activity, negative values indicate that BA17 drives rLO activity. Bars indicate ± one standard error of the mean for repeated measures, *p*-values indicate results of paired *t*-tests or *F*-tests for repeated measures. a. u., arbitrary units.

The PSI values for contour and non-contour conditions in the contour detection task are shown in Figure 6A. Red color indicates an inflow of information from rLO into BA6, IPL, and BA17, respectively, and blue color indicates an outflow of information. Whereas some information flow was observed in each pairing and condition, a significant PSI difference between contour and non-contour conditions was present only for the pairing between BA17 and rLO (150 connected bins, *p* = 0.003 by permutation test; Figure 6B). The difference was significant between 17 and 26 Hz, in a time range from 20 to 340 ms. The strongest differences occurred early after stimulus onset (~80 ms) and between 200 and 320 ms. In both time ranges, the PSI had a positive value in the contour condition and a negative value in the non-contour condition. This indicates that the communication between rLO and BA17 during the contour condition was characterized by a flow of information from rLO to BA17, whereas in the non-contour condition it was mainly characterized by a flow from BA17 to rLO.

In order to see whether the difference between the BA17—rLO PSI for contour and non-contour conditions was specific for the contour detection task, it was compared with the corresponding PSI in the luminance change detection task. To this end, the PSI within the significant time-and-frequency bins as highlighted in Figure 6B was averaged across subjects, within the contour and non-contour conditions of both tasks. The results are depicted in Figure 6C. The PSI difference between contour and non-contour conditions was significant in the contour detection task, *t*_(17)_ = 5.46, *p* < 0.001. A similar difference was not observed for the luminance detection task where contour as well as non-contour stimuli produced a moderate flow of information from rLO to BA17, *t*_(17)_ = −0.23, *p* > 0.8. The interaction between stimulus type (contour, non-contour) and the task (contour detection, luminance-change detection) was significant, *F*_(1, 17)_ = 8.47, *p* = 0.010.

#### Individual differences and behavioral correlates

Individual differences were obtained for three measures where the group-level differences between contours and non-contours were significant. ERP differences were investigated on the mean amplitude 168–188 after stimulus onset, at electrode P6 where the largest posterior difference occurred (see Figures 2A,D). Beta power differences in rLO source activity were obtained for the mean power 160–220 ms after stimulus onset, at 14–16 Hz (see Figure 4C). Finally, PSI differences in the BA17-rLO pairing were investigated. The PSI values were averaged per subject across significant time and frequency bins as shown in Figure 6B. For all measures, activity in the non-contour condition was subtracted from that in the contour condition.

As a result, 12 out of 18 subjects showed larger (more negative) N1 amplitude in the contour compared to the non-contour condition. This is a larger number of subjects than can be expected by chance, given an a priori probability of 0.5 (*p* = 0.048 by binomial test). Thirteen out of 18 subjects showed larger beta power in rLO within the investigated time range (*p* = 0.015). Moreover, 17 out of 18 subjects showed a more positive PSI in the contour compared to the non-contour condition for the BA17—rLO pairing (*p* < 0.001). The results together suggest that the group-level effects also consistently occurred within participants.

**Figure 7:**
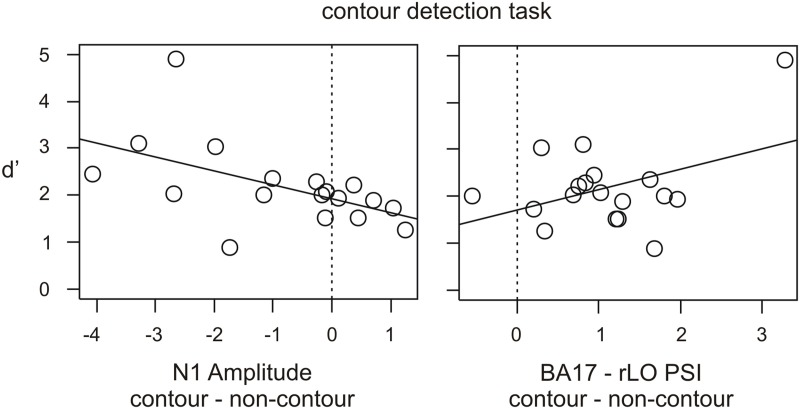
**Scatter diagrams showing individual differences in the N1 amplitude and PSI value in contour minus non-contour conditions (contour detection task), and the relation with *d*' as a measure for the behavioral performance**.

Finally, the obtained individual differences in N1 amplitude, rLO beta power and BA17—rLO PSI were correlated with the behavioral data. The reaction times and hit rates did generally not correlate with the physiological measures (all *p* > 0.112). In contrast, the *d*' values showed a significant negative correlation with N1 amplitude differences (*r* = −0.536, *p* = 0.022) and a corresponding positive trend for PSI differences (*r* = 0.414, *p* = 0.088). The results are depicted in Figure 7. Across subjects, larger (more negative) N1 amplitudes in the contour compared to the non-contour condition were associated with higher sensitivity scores (*d*'). Similarly, a more positive BA17—rLO PSI in the contour compared to the non-contour condition predicted a larger *d*'. The latter result was strongly influenced by a single participant with large *d*' and PSI values. In order to control the effect of outliers, Spearman rank-correlations were additionally computed. The results showed a significant rank-correlation between N1 amplitudes and *d*' values (rho = −657, *p* = 0.004), but not between the BA17—rLO PSI and *d*' (rho = −0.01, *p* > 0.9). Thus, there was no evidence for a correlation in the latter case.

## Discussion

We tested the hypothesis that contour integration requires a top-down selection, rather than a passive pooling, of neural information coding the elements of a contour. To this end, the participants were presented with contour and non-contour displays, where the contour was either relevant (contour detection) or not relevant (luminance-change detection) to the experimental task. The results showed larger N1 amplitudes in contour compared to non-contour conditions, reflecting an increased LO activity in that time range as reconstructed from ERP source estimation. Importantly, the difference was restricted to the task where contour integration was required. This situation was also characterized by an increased inflow of information from LO to primary visual areas during contour processing. These results are consistent with the view that contour integration requires a selection of information from lower visual areas, and that this selection is initialized from lateral occipital regions.

Our ERPs and their sources in the contour detection task largely replicated and confirmed previous findings on contour processing. The earliest significant differences were observed during the N1 latency, and were most prominent during the peak of that component. Corresponding differences in the N1 time range have been reported in most EEG and MEG studies on contour integration (Mathes et al., 2006; Machilsen et al., 2011; Pitts et al., 2012; Shpaner et al., 2013; cf. Tanskanen et al., 2008). The topography of the N1 difference is also similar. Our difference topography at the N1 peak shows a bipolar current distribution with a negativity at right temporo-parietal electrodes and a corresponding positivity at left central and frontal sites. Although in some previous studies the ERP difference was more focused at parietal sites (e.g., Tanskanen et al., 2008; Machilsen et al., 2011) the pattern of a bipolar frontal and posterior topography matches closely with the topography in our study.

Furthermore, the neural source of the N1 amplitude difference, as obtained from a distributed source imaging, is also in good agreement with the results from previous studies where source estimations were applied. The largest differences in the estimated sources in our study occurred in the lateral part of the right medial occipital cortex, rLO. Tanskanen et al. (2008) found differences between event-related fields in contour and non-contour conditions in a similar time range, with reconstructed sources in parieto-occipital regions, including middle and superior occipital gyrus as well as precuneus. Other authors report source estimates in the more lateral parts of the left and right occipital complex (Shpaner et al., 2013). Some studies also revealed active sources in lower visual cortex during the N1 or P2 time range, as estimated from the ERP. For example, Pitts et al. (2012) obtained focal activity in bilateral primary visual cortex during the processing of highly salient contour patterns. Also Shpaner et al. (2013) report that, while the largest differences occurred in lateral occipital regions, the sources reconstruction revealed small patches of activation within early visual cortex. It is noteworthy that the lateral occipital focus estimated in our and in previous EEG studies fit well with findings from functional magnetic resonance imaging (fMRI). Kourtzi et al. (2003) used an fMRI adaptation task where an adapting stimulus with randomly oriented Gabor patches or lines changed to either a different random orientation pattern or to an array containing a contour path. The rebound of activity in the test period was larger in the latter case, suggesting that neural activity was enhanced in the presence of a contour. This enhancement was most prominent in the lateral occipital complex (see also Altmann et al., 2003, 2004).

Using source level analyses and rLO as a seed region, we identified a broad brain network with hubs in IPL, BA6, and BA17. The network was defined by correlated activity in a small spectral component of the source signal within the beta frequency range (~15 Hz). In line with previously published results by our group, a corresponding beta power difference between contour and non-contour conditions was also found at the EEG channel level in occipito-parietal regions (Volberg et al., 2013b). The current results demonstrate that beta oscillations also differ in the source signals obtained for contours and non-contours, thereby again emphasizing the special role of beta oscillations for contour integration. Another interesting finding obtained from power envelope correlations is that rLO was not only functionally connected to early visual areas, but also to more dorsal areas IPL and BA6. Area BA6 is traditionally considered to be a pre-motor area, but is also involved in various cognitive operations. Specifically, the lateral parts of BA6 seem to have a critical role in updating the internal spatial representation of external stimuli (Tanaka et al., 2005). It is reasonable to assume that perceptual groupings in LO change the spatial representation of the whole scene, which must then be updated accordingly. This would be a possible role of BA6 within the beta network.

The role of IPL in contour integration has been more intensively investigated. In a recent study, we examined how rhythmic changes in brain connectivity affect the probability for detecting contours in similar displays (Hanslmayr et al., 2013). It turned out that the detection performance depended on the bidirectional information flow between IPL and LO. If a contour was presented during highly connected states with strong information flow, then the target was more likely to be detected compared to a state of low connectivity and weak information flow. This suggests that a spatial reference, provided by the parietal brain, aids the integration of contours within lateral occipital areas (Robertson, 2003; Mevorach et al., 2006). Taken together, the roles of BA6 and IPL in the beta network might both be related to spatial operations, i.e., providing a spatial reference for contour elements or updating the spatial representation of the global contour within working memory. Further studies are required to elucidate these different options.

Most interesting for the purpose of the present study, there were no differences in the brain activity between contour and non-contour conditions in the luminance-change detection task. We observed no difference in the ERP N1 amplitude, no difference in the estimated LO activity, and no difference in the beta band response, all of which are demonstrably present in contour detection task when the contour is task-relevant (Tarokh, 2009; Shpaner et al., 2013; Volberg et al., 2013b). The outcome broadly supports the notion that contour integration is not an automatic process, but rather requires a selection of information from lower brain regions.

It should be noted that in some previous studies differences between contour and non-contour conditions were also observed during passive viewing tasks (Tanskanen et al., 2008; Machilsen et al., 2011) or with perceptual adaptation tasks (Kourtzi et al., 2003). This seemingly contradicts our proposition that contour integration relies on top-down selection. Indeed, top-down control is especially important in situations where the bottom-up grouping cues are weak and the saliency of the contour is relatively low. The saliency depends on different task and stimulus factors like the local path angle (e.g., Field et al., 1993), the length of the contour (Li et al., 2006, 2008), the spacing between the elements making up the contour (Li and Gilbert, 2002; Beaudot and Mullen, 2003; Roudaia et al., 2013), or the location within the visual field (Volberg, 2014). Closure is a further strong grouping cue (Kovács and Julesz, 1993; Donderi, 2006). In all previous studies with a passive viewing task, such highly salient closed contours were used, possibly favoring a more automatic selection and thus producing differences between contour and non-contour conditions without top-down control. Similar results can be expected with other highly salient contours.

Furthermore, it is possible that previously less salient contours become ever more salient while practicing the contour detection task. As a consequence, contour detection and the associated differences in brain activity between contour and non-contour stimuli would require progressively less top-down control (Fahle and Poggio, 2002; Ahissar and Hochstein, 2004). For example, whereas in Li et al. (2008) contour and non-contour stimuli task did not produce differential activity in a luminance change detection task before training, they did so after practicing contour detection. A similar pattern of results might also occur in an EEG experiment if the luminance change detection task is investigated before and after practice. Nonetheless, and more important for our study aims, we found no differences in brain activity between non-contours and less salient contours before training. This observation suggests that the integration of these contours is not solely a stimulus-driven process.

As a further critical point, our luminance change detection task required the participants to attend to the screen center, whereas in the contour detection task the whole stimulus display should be attended. It may thus be objected that task-related differences between contour and non-contour conditions occurred because the contours were unattended in the former task and attended in the latter one. This explanation cannot be excluded from the data. On the other hand, the few available studies on the role of spatial attention in contour integration seem to suggest that these two processes are partly independent (Roelfsema et al., 2010; Vancleef et al., 2013). Moreover, it is known that the enhancement of neural activity induced by spatial attention is inversely related to the size of the attended region (Müller et al., 2003). Because the participants had to attend to the whole display during the contour detection task, a possible facilitation of contour integration by spatial attention would be relatively low. Together it seems unlikely that differences in spatial attention alone can account for the observed differences between contour and non-contour conditions in the two tasks.

Our finding that contour and non-contour stimuli did not produce differential brain activity in the first blocks of the experiment where the observers were unaware of the contours complies with the results of Li et al. (2008) obtained with untrained or trained monkeys with the same paradigm. Our results suggest that, at least if the resulting shape is unknown in advance, contour integration involves top-down selection.

As the novel contribution of this study, our PSI results also suggest where and how the selection takes place. Because this is the first report on PSI differences in contour and non-contour processing, the results should be taken as preliminary and can only be interpreted with caution. A possible interpretation could read as follows. Consider that a contour stimulus is presented to an observer. Collinear elements would be identified during the bottom-up sweep, and lateral connections between the coding neurons would be enabled (Roelfsema, 2006). If a neuron in higher visual areas is tuned to that particular shape, then a contour grouping can be achieved within the same bottom-up sweep. If such stimulus-driven grouping is not possible, all further processing would depend on whether the contour is relevant for perception or action. In situations where they were task-relevant, contour stimuli produced a strong inflow of information from rLO into primary visual areas.

Within early visual cortex, the activity would propagate through the network that was established during the bottom-up sweep. In this way, a population of neurons coding the elements of a common object can be separated from other neural activity, thereby making the perceptual group accessible. In contrast, the information flow in the non-contour condition was from primary visual areas into LO, possibly reflecting the persistent signaling of contour candidates into higher visual areas. Importantly, the differences in the directional flow of information between BA17 and rLO for contour and non-contour stimuli was completely absent in the luminance change detection task where the contour was not task relevant. In line with the predictions drawn from the IGT, the results show that contour grouping, at least with less salient contours, is achieved through feedback into early visual cortex. Moreover, they show that the feedback is initialized from lateral occipital regions.

It is not clear at present whether feedback from LO into lower visual regions only occurs during the grouping of contour elements into previously unknown shapes, or whether it also occurs in a stimulus-driven grouping of pre-defined shapes. Altmann et al. (2003) presented closed contours in two different tasks and found a similar increase of brain activity in LO, but a differential increase in early visual areas compared to non-contours. The fact that lower visual cortex activity depended on the task suggests that feedback from LO is also provoked if the contour grouping is driven by the stimulus. However, a closer examination with different types of contour stimuli would be necessary to clarify this point. Given that LO exerts top-down feedback during contour grouping, it should also be mentioned that the data allow for two different interpretations. According to the IGT, the feedback would enhance the activity of single elements that belong to the same perceptual group. A different possibility is that top-down control does not affect the elements per se, but the lateral interactions between them (Freeman et al., 2001, 2003). Unfortunately, with the present data set we cannot contribute to this controversy, so that the question concerning whether top-down control affects the elements or the linking between the elements of a perceptual group needs to be left open for future research.

By considering that the task and the stimulus type affected the N1 amplitude, the beta source power, and the PSI in a similar way, one might ask whether these measures reflect the same neural mechanism. This seems very unlikely, since the measures reflect very different aspects of ongoing brain activity. On the one hand, contour compared to non-contour conditions produced a focal increase in brain activity around 168–188 ms after stimulus onset, with sources estimated within the right lateral occipital complex. At the same time, this brain region showed an increased inflow of information into primary visual areas (BA 17) during contour compared to non-contour conditions. Moreover, both regions were part of a broader network defined by co-variations in the beta frequency range. By investigating brain oscillations in the source space, we could describe long-range neural communication during contour and non-contour processing, in addition to the temporal and spatial characteristics of focal neural activity differences as reflected in the ERP and the associated source estimate.

In summary, we investigated the neural mechanisms of contour integration in two different situations where the contour was either relevant or not relevant for the task. Only in the former condition we found differential brain activity between contour and non-contour conditions, within a distributed network including estimated parietal, lateral occipital and primary visual areas. In this situation, contour processing was associated with an inflow of information from lateral occipital into primary visual regions. The findings suggest that contour integration results from a selection of task-relevant information from early visual areas, and that this selection is driven by the lateral occipital cortex. The results of future studies might extent this finding to other stimulus or task conditions.

### Conflict of interest statement

The authors declare that the research was conducted in the absence of any commercial or financial relationships that could be construed as a potential conflict of interest.
