# Supplementary material for: Brain networks supporting perceptual grouping and contour selection
Source: Front Psychol. 2014 Apr 4;5:264. doi: 10.3389/fpsyg.2014.00264 (PMC3983489; doi:10.3389/fpsyg.2014.00264)
Supplement: Supplementary file 1 [file Presentation1.PDF]

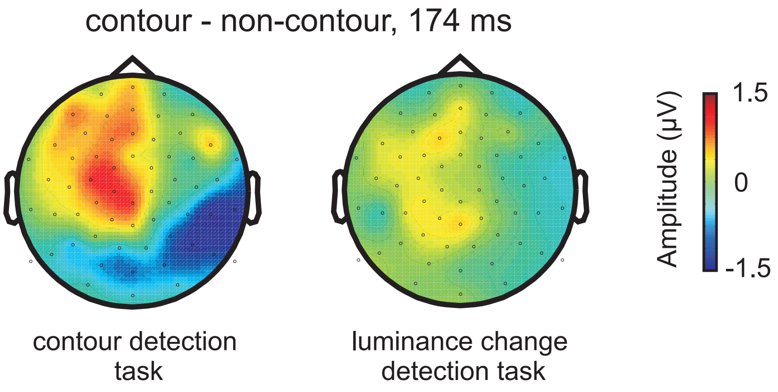

Supplemental Figure 1: Head topographies of ERP difference waves, contour minus non-contour condition, at the N1 peak difference. The data were re-references to an average reference value and show a more posterior difference in the contour detection task, compared to the head topographies shown in Figure 2 where a mastoid reference was used.
